# Mechanistic insights into nanoparticle surface-bacterial membrane interactions in overcoming antibiotic resistance

**DOI:** 10.3389/fmicb.2023.1135579

**Published:** 2023-04-21

**Authors:** Suraj Kumar Modi, Smriti Gaur, Mrittika Sengupta, Manu Smriti Singh

**Affiliations:** ^1^Department of Biotechnology, Bennett University, Greater Noida, Uttar Pradesh, India; ^2^Centre of Excellence for Nanosensors and Nanomedicine, Bennett University, Greater Noida, Uttar Pradesh, India

**Keywords:** nanomedicine, AMR, antibiotic, membrane, resistant

## Abstract

Antimicrobial Resistance (AMR) raises a serious concern as it contributes to the global mortality by 5 million deaths per year. The overall impact pertaining to significant membrane changes, through broad spectrum drugs have rendered the bacteria resistant over the years. The economic expenditure due to increasing drug resistance poses a global burden on healthcare community and must be dealt with immediate effect. Nanoparticles (NP) have demonstrated inherent therapeutic potential or can serve as nanocarriers of antibiotics against multidrug resistant (MDR) pathogens. These carriers can mask the antibiotics and help evade the resistance mechanism of the bacteria. The targeted delivery can be fine-tuned through surface functionalization of Nanocarriers using aptamers, antibodies etc. This review covers various molecular mechanisms acquired by resistant bacteria towards membrane modification. Mechanistic insight on ‘NP surface-bacterial membrane’ interactions are crucial in deciding the role of NP as therapeutic. Finally, we highlight the potential accessible membrane targets for designing smart surface-functionalized nanocarriers which can act as bacteria-targeted robots over the existing clinically available antibiotics. As the bacterial strains around us continue to evolve into resistant versions, nanomedicine can offer promising and alternative tools in overcoming AMR.

## 1. Introduction to AMR

The emergence of antimicrobial resistance is rendering antibiotics ineffective for an ever-increasing number of infections. World Health Organization (WHO) reported in 2019, the pipeline of 32 new antibiotics against the priority pathogens, amongst which only 6 of them were found to be novel. A study published in The Lancet 2022, suggests 4.95 million mortalities can be attributed to cases concerned with resistant bacterial infections (Murray et al., 2022). Out of which, 1.27 million fatalities were solely due to AMR. Hence, antimicrobial resistance poses a global threat and requires concerted actions with immediate effects by the healthcare community and the policy makers. Bacterial resistance arises mainly due to the lack of proper stewardship of the available drugs. The broad range antimicrobials generally prescribed to treat wide spectrum of bacterial infections and the ease of availability of the drugs became the root cause of drug resistant infections. Till date, the pace of discovery and approval of novel therapeutics is a limiting factor.

WHO enlists a category of fatal pathogens as ESKAPEE which includes *Enterococcus faecium*, *Staphylococcus aureus*, *Klebsiella pneumoniae*, *Acinetobacter baumannii*, *Pseudomonas aeruginosa*, *Enterobacter* spp., and *Escherichia coli* species reported to have become MDR pathogens. Broadly, the antibiotic-sensitive strains undergo genomic mutation or acquire antibiotic-resistant genes through horizontal gene transfer. These strains result in pathogenic MDR phenotype and the culprit behind most nosocomial infections. Plenty of clinical data into the mechanisms of antibiotic resistance obtained from isolated pathogenic microorganisms across hospitals is being reported routinely (Wang et al., 2020b; Murray et al., 2022). Hence, it is imperative to thoroughly explore potential targets both at the genotypic and phenotypic level. Membrane-based targets being the first line of defence should be closely investigated, especially in ESKAPEE subgroup as they have the potential to respond towards novel therapeutics and reverse AMR.

Emerging technologies such as nanomedicine have potential as alternative therapeutics in mitigating AMR. Nanoparticles exhibit innate anti-bacterial potential or can serve as a ‘Trojan-horse’ to deliver antibiotics to drug resistant bacteria. Antibiotics administered in bulk can cause tissue-toxicity, but is administered nonetheless in increased doses to mitigate the pathogen and achieve its therapeutic effect. At the physiological level, utilizing nanocarriers as antibiotic-delivery vehicle have shown promising outcomes mainly due to improved pharmacokinetics in terms of lowering the volume of distribution of antibiotic at the site of infection and extending serum half-life due to slow antibiotic release. These nanocarriers can be further improvised by decorating their surface with molecules like antibodies, aptamers etc. specific to targets on the bacterial membrane and reduce the undesirable systemic toxicity in patients.

This review covers the different mechanisms used by the MDR pathogens for membrane modification. In our research for this review article, we came across few articles highlighting the mechanism of bacterial death. Therefore, we have mainly included recent articles which elaborated on the mechanistic insights into bacterial membrane-nanoparticle surface interaction. We believe, this can have implications in establishing the significant role of NPs as an alternative or synergist to antibiotic therapy. Since the bacteria continue to develop resistance to the available antibiotics, nanomedicine can prove to be a boon as a targeted molecular therapy in combating MDR pathogens.

## 2. General mechanism of membrane-mediated AMR

**Figure 1:**
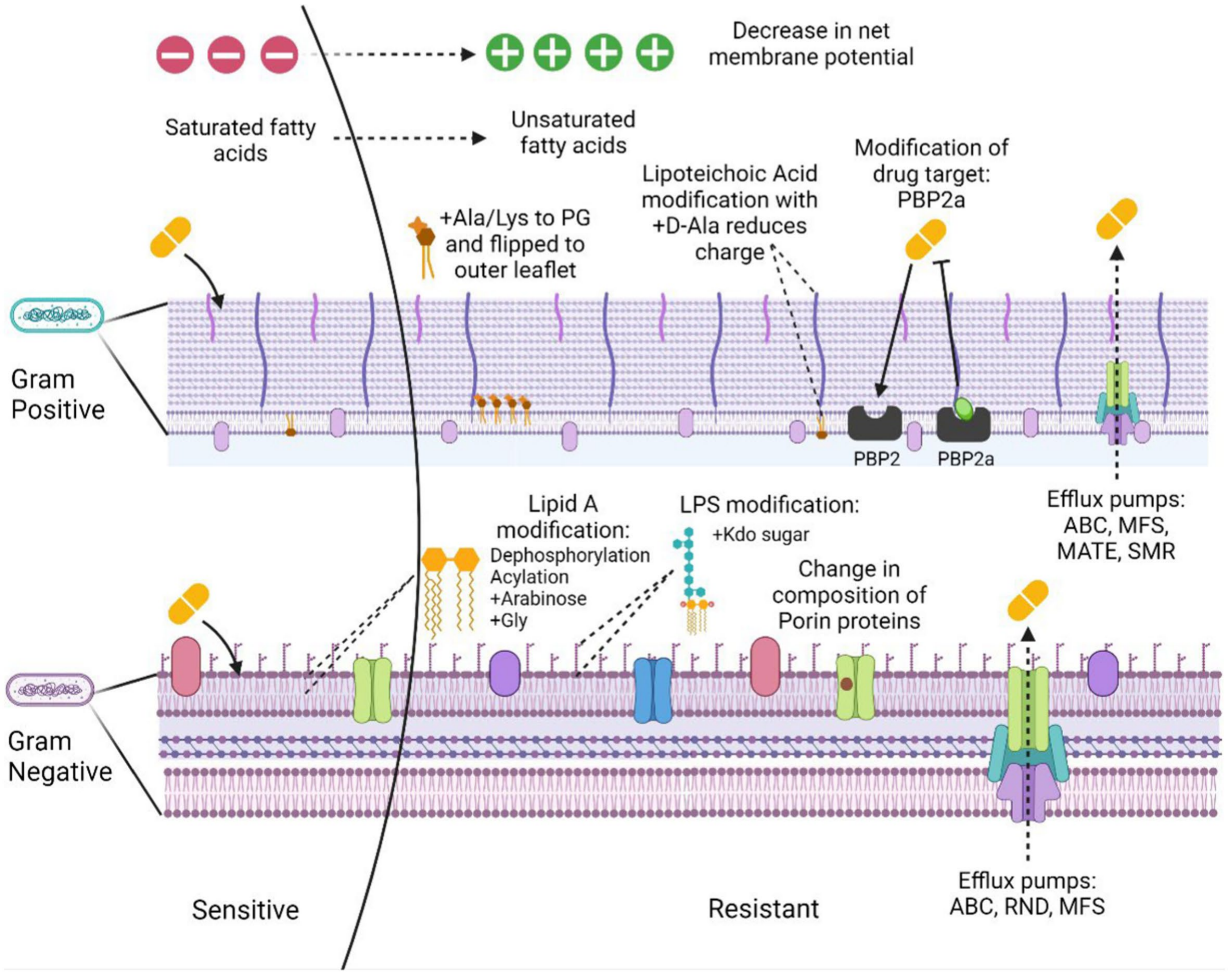
Mechanisms of membrane-based drug resistance in Gram positive and Gram negative bacteria. Figure created in Biorender.

Antimicrobial resistance is the ability of the bacteria to resist the action of an antimicrobial hence making the treatment unsuccessful. The problem is aggravated due to the ability of many pathogenic bacteria to form biofilms and the presence of persister cells in the population. These cells occupy around 1% of the bacterial population in the culture that is in the dormant phase (Keren et al., 2004; Wood et al., 2013). The bacterial resistance mechanisms could be intrinsic, i.e., the inherent nature of some bacterial species to possess functional or structural modifications providing resistance to the antibiotic. Acquired resistance, however, is the resistance developed over time from selection pressure or through horizontal transfer from other bacterial strains. The overall resistance mechanisms can be categorized as: (a) limiting drug uptake, (b) target modification, (c) drug inactivation, and (d) activated efflux pumps (Figure 1).

(a) Limiting drug uptake

The limited drug uptake arises due to certain structural components of the Gram-positive and the Gram-negative bacterial cell walls that provide a barrier to the uptake of many antimicrobial agents. The presence of an additional outer layer with increased lipid content in case of mycobacteria, allows the passage of hydrophobic drugs with ease but limits the access of hydrophilic drugs (Lambert, 2002). Gram negative bacteria possess an additional outer membrane that permits the passage of the hydrophilic drugs through β-barrel protein channels called porins (Gill et al., 1998; Blair et al., 2014). Drug uptake through these channels is often restricted by downregulation of these porins. Recently, it was identified that *K. pneumoniae* mediates resistance against carbapenem, considered as a drug of last resort, via mutations that constrict the outer membrane porins (OmpK35) and (OmpK36; Wong et al., 2019) and restrict uptake of the antibiotic.

(b) Target modification.

Of the most common modifications used by the Gram-positive bacteria towards the beta lactam antibiotics are the variation in the penicillin binding proteins (PBPs). The altered PBP2a protein acquired by methicillin resistance gene (*mecA*) present on Staphylococcal cassette chromosome mec (SCCmec) mobile genetic element in *S. aureus* (Reygaert, 2009) confers resistance to methicillin. Vancomycin resistance is acquired through *van* genes, resulting in the altered metabolites of peptidoglycan synthesis, causing decreased binding to the antibiotic (Beceiro et al., 2013; Cox and Wright, 2013).

(c) Drug inactivation.

Yet another mechanism of resistance is through drug inactivation that occurs in majorly two ways: by complete degradation or by surface modification. Actual degradation of a drug can be done through hydrolyzing enzymes such as beta-lactamase. There are over 7,000 characterized β-lactamases. Carbapenamases such as *K. pneumoniae* carbapenemase (KPC), New Delhi metallo-β-lactamase (NDM) and oxacillinase (OXA) are known to inactivate multiple antibiotics such as cephalosporins and penicillins apart from carbapenems (Queenan and Bush, 2007). However, tetracycline class of antibiotic can be inactivated through *tetX* gene (Kumar et al., 2013; Blair et al., 2015). TetX catalyzes resistance through oxygen mediated drug destruction, depending on flavin adenine dinucleotide (FAD) for its monooxygenase activity (Yang et al., 2004). Bacteria also produce enzymes that are capable of altering the drug molecule. Enzymes such as phosphotransferases can modify drugs such as aminoglycosides and macrolides (Golkar et al., 2018).

(d) Activated efflux pumps.

Other category includes the genes encoding efflux pumps, which are present on the bacterial chromosomes. Some of these genes exhibit constitutive expression while others are induced through a stimulus or a specific substrate. The primary purpose of these efflux pumps is to eject out the toxic substances out of the cell (Blair et al., 2014). Some recent studies in resistant *Acinetobacter nosocomialis*, showed that AdeRS two component system (TCS) confers resistance to tigecycline, eravacycline antibiotics through Ade ATP binding cassette (AdeABC) efflux pump (Lee et al., 2020) therefore, AdeRS system can be a prospective drug target against tigecycline resistance. Similarly, these broad-spectrum antibiotics of last resort have also been targeted in the Gram-positive Staphylococcus species, through a variation in Tet(L) efflux pump leading to a compromise in the effective treatment with these drugs (Wang et al., 2021). Other significant impact of resistance to tetracyclines and ciprofloxacins is through CmeABC efflux pump in *Campylobacter jejuni*. The *cmeA* gene was found to be highly expressed in tetracycline resistant isolates hence, conferring resistance (Sharifi et al., 2021). Evolution has paved a way for many efflux pumps to become major mediators of antimicrobial resistance.

## 3. Resistance via bacterial membrane modification

Bacterial membranes form an accessible target for majority of the antimicrobials. The outer membrane in Gram-negative bacteria performs a vital role in forming an additional protective layer without compromising on the exchange of components important for sustainability. The modification of the lipid bilayer with certain porin proteins of specific size makes it a permeability barrier. The impact of this lipid bilayer is crucial to the antibiotic susceptibility of the microorganisms. The hydrophilic drugs like beta-lactams, make use of pore formation through porin proteins to breach and enter the cell whereas, the hydrophobic drugs including the macrolides diffuse across the cell. Following are the kinds of membrane alterations responsible for acquiring resistance:

### 3.1. Membrane remodeling

In Gram-negative bacteria like *E. coli* the overall composition of the membrane can be altered by degrading the existing proteins and incorporating the molecules into new proteins present in the outer membrane. Certain proteases present in the outer membrane such as BepA, DegS, YcaL degrade the damaged proteins (Chang, 2016; Daimon et al., 2017; Soltes et al., 2017). At the level of transcription, porin encoding genes are controlled by two-component systems, non-coding RNAs and other regulatory molecules. These systems work under the regulatory control of environmental stimuli (Pratt et al., 1996; Chen et al., 2004; De La Cruz and Calva, 2010) hence, working at the systemic level. Upon sensing the environmental osmolarity or the ethanol levels, *EnvZ*-*OmpR* TCS gets triggered to re-establish the composition of porin proteins present in the outer membrane (Fernández and Hancock, 2012; Chang, 2016; Kenney and Anand, 2020). *CpxR* member of the envelop stress response also sense the environmental osmolarity or the antimicrobials (Raivio, 2014; Delhaye et al., 2016). Treatment with the drug increases the levels of *micC* (small non-coding RNA) which work in concert with the *cpxR* gene to remodel the bacterial membrane against the β-lactams (Dam et al., 2017).

### 3.2. Lipid glycosylation using amine-sugars

The positively charged sugar residues attach to the lipidA molecules present as a component of LPS in the bacterial membrane, thus neutralizing the negative charge conferred by the phosphate groups. Such modifications prevent the binding of cationic antimicrobials leading to resistance. One such example was identified in resistant Gram-negative *Salmonella typhimurium, E. coli* and *P. aeruginosa* species, where 4-amino-4-deoxy-l-arabinose (l-Ara4N) moiety having positively charged amine group at C-4 position is attached to the negatively charged phosphate group of lipidA(C-4; Trent et al., 2001; Zhou et al., 2001). The *phoPQ* TCS in Salmonella transcribes *pmrCAB* locus conferring polymyxin resistance. This system controls activation of genes responsible for modifying lipidA with Ara4N (Trent et al., 2001; Zhou et al., 2001; Zavascki et al., 2007). A recent study identified *pmrAB* system in *P. aeruginosa* that is involved in lipidA modification through Ara4N (Moskowitz et al., 2004). Besides arabinose modification, galactosamine residues were also found to be responsible for developing resistance to the antibiotics. Such as in case of *A. baumannii* with colistin resistance features a D-galactosamine attachment to lipidA 1-phosphate residue (Pelletier et al., 2013).

### 3.3. Membrane lipid modification via amino acids

Human colonizing pathogens such as *Staphylococcus aureus* acquires resistance to Cationic antimicrobial peptides (CAMPs) by expressing *mprF* (multiple peptide resistant factor; Ernst and Peschel, 2011). *mprF* catalyzes the addition of Ala or Lys residues on phosphatidyl glycerol moieties. This leads to the transition of charge on the phospholipid to positive with the addition of lysine or neutral with alanine. The other domain of *mprF* is a flippase that flips the peptidoglycan alanine (PG-Ala) and peptidoglycan lysine (PG-Lys) residues to the outer membrane (Ernst et al., 2009; Klein et al., 2009). This translocation of the charged residues reduces the overall negative charge on the membrane and hence, decrease the binding affinity of the cationic antibiotics. The decrease in the affinity of the positively charged membrane disintegrants can also arise from the incorporation of D-alanyl residues mediated via the *dlt* operon (Neuhaus et al., 1996; Kovács et al., 2006). GraSR, TCS overexpresses the *dlt* operon system, which is subjected to resistance development in *Staphylococcus aureus* to anionic daptomycin (Li et al., 2007a; Yang et al., 2009). In a recent study, the resistant *Vibrio cholerae* to polymyxin B was found to alter lipidA via glycine amino acids on the acyl chain residue of glucosamine (Hankins et al., 2012; Henderson et al., 2014).

### 3.4. Addition of phosphoethanolamine

LipidA modification with pEtN residue on 1-phosphate, leads to resistance in Gram negative pathogens. This surface change brings upon positive charge to the lipidA while lowering the interaction of positively charged antibiotics to the membrane. This change is brought about by *pmrC* enzyme which works under the stringent control of *pmrAB* TCS. Colistin resistance in *A. baumannii* arises due to the modification of lipidA group by pEtN residues (Pelletier et al., 2013). Additionally, pEtN can also be incorporated on LPS kdo sugar subunits of Gram-negative *E. coli* and Salmonella species (Kanipes et al., 2001; Raetz et al., 2007).

### 3.5. Efflux pumps

Efflux pumps are membrane transporters which work to expel out smaller molecules like antibiotics which targets the intracellular organelles/ biomolecules, hence developing resistance to them. The ATP binding cassette (ABC) transporters, and membrane bound efflux pumps, couple the energy of ATP hydrolysis to expel out the substrate. *VraFG* transporter promotes resistance in *S. aureus* and *Staphylococcus epidermidis* species to CAMP (Li et al., 2007a,b). In *S. epidermidis* expression levels of the homologues of *vraFG* are high in presence of human cationic antimicrobial peptide human beta defensin 3 (hCAMP hbD3) under regulation of the *aps* operon (Li et al., 2007b). Whereas *S. aureus* expresses *vraFG* system under high levels of hCAMP stress (Li et al., 2007a) resulting in resistance against these CAMPs besides the anionic antimicrobials or neutral peptides (Li et al., 2007a,b). Alternately, these ABC transporters permits the passage of the antimicrobials inside the cell for them to get degraded by the proteases (Li et al., 2007b). Such mechanism was reported in *S. typhimurium* for melittin / protamine residues.

### 3.6. Remodeling lipid acyl tail

Remodeling of the acyl group of lipids leads to modification in membrane thickness and fluidity, which aids in developing resistance to various antimicrobial agents as has been seen in bacteria such as *Enterococcus faecalis* (Aricha et al., 2004; Kumariya et al., 2015; Maria-Neto et al., 2015). Such alterations in the bacterial membrane lipids include modification in the content of unsaturated fatty acids, the length and proportion of the acyl tail that incorporates the branched tail lipids and cyclopropane (Sohlenkamp and Geiger, 2016; López-Lara and Geiger, 2017). Previous studies suggest, that a less branched lipids in the bilayer membrane having a long and saturated chain assists in forming a thick and ordered lipid structure along with slow diffusion of lipids (Filippov et al., 2007; Kučerka et al., 2011; Poger et al., 2014; Marquardt et al., 2016; Levental et al., 2020). Such changes in the lipid composition of bacteria leads to development of resistance against the antimicrobial peptides (AMPs). For example, increasing unsaturated lipid moieties potentially inhibit the construction of daptomycin oligomers followed by varied pore formation by daptomycin (Taylor et al., 2017; Beriashvili et al., 2018). Similarly, Tao et al., 2021 reported that Gram negative bacteria exhibited membrane plasticity and suggested significant impact on glycerolipids in response to colistin treatment (Tao et al., 2021). Apart from modifications in the physio-chemical properties of the membrane, remodeling of acyl tail can occur in response to change in enzymatic pathways which ultimately develops resistance (MacDermott-Opeskin et al., 2022). For example, Hines group reported that pgsA mutation resulted in reduced PG content, aggregation of fatty acids upstream and successive reassembling the profiles of fatty acids (Hines et al., 2017).

## 4. Potential membrane targets to facilitate drug targeting

The presence of the peptidoglycan layer on the bacterial cell wall, as well as other unique features such as specific ion channels and proteins that are not found on the host cells make the bacterial cell wall a preliminary target to the available therapeutic options. Over the years bacteria have evolved ways to develop resistance to almost every available antimicrobial. It has been anticipated that AMR will emerge to be one of the leading causes of death by 2050 if the problem is not dealt with immediate effects (O’Neill, 2016). To facilitate drug targeting, bacterial membrane provides a platform for directed interaction of a metabolite in the form of a protein, enzyme, or any surface receptor to that of a drug. Table 1 summarizes the list of potential drug targets present in the resistant ESKAPEE pathogens.

**Table 1:** Potential targets for resistant ESKAPEE pathogens.

Location	Gene	Role/function	Organism	References
Cell Membrane	*efrAB*	ABC transporter, Tolerance and resistance to biocides	*E. faecium*	Lavilla Lerma et al. (2014)
	Genes responsible for encoding LPS-O-antigen	Bacterial capsule retention, immunogenic	Resistant *K. pneumoniae*	Singh et al. (2022)
	*bfmS*	TCS sensor kinGase, Production of outer membrane vesicles	*A. baumannii*	Kim et al. (2019)
	*amgS*	Function as a part of envelope stress response to aminoglycoside induced abberant polypeptides	Resistant *P. aeruginosa*	Ho-Fung Lau et al. (2013)
	*mphE* and *msrE*	Macrolide resistance genes. mphE encodes macrolide-2′-phosphotransferase. msrE belongs to ABC-F subfamily of ATP binding cassett protein	Resistant *P. aeruginosa*	Chen et al. (2020)
	*mexAB-oprM*, *mexCD-oprJ*, and *mexXY-oprM*	Efflux pumps	Resistant *P. aeruginosa*	Azimi et al. (2015)
Periplasmic	Fe+2 Enterobactin ABC transporter substrate binding protein	Iron enterobactin transporter	Carbapenem resistant *K. pneumoniae*	(Mehmood et al., 2020)
Outer membrane	*ompK37*	Outer membrane porin protein N	Carbapenem resistant *K. pneumoniae*	Mehmood et al. (2020)
	*ompA*	Virulence factor, mediate biofilm formation, Eukaryotic cell infection, immunomodulation	*A. baumannii*	Nie et al. (2020)
	*carO*	Responsible for cell adherence and virulence	Carbapenem resistant *A. baumannii*	Labrador-Herrera et al. (2020)
Outer Membrane protein	*mipA* (mltA interacting protein)	Kanamycin resistance	*E. coli*	Zhang et al. (2015)
Membrane proteins	*acrAB-tolC* efflux pump and bla_NDM-1_	Carbapenem resistance	*E. coli*	Chetri et al. (2019)
Mobile Genetic element SCCmec	*mecA*	Methicillin Resistance	*Methicillin resistant Staphylococcus aureus (MRSA)*	Jevons (1961), Katayama et al. (2000), Olsen et al. (2006)
Van operon present on transposons/ chromosomes	*vanA*	Vancomycin resistance	*Vancomycin resistant Staphylococcus aureus (VRSA)*	Arthur et al. (1993) and Périchon and Courvalin (2009)
On Chromosome encoded by plasmid	*optrA*	Resistance to oxazolidinones	*E. faecium*	Wang et al. (2015)
	*ISEcp1-bla_CTX-M_*	AMR	*E. cloacae*	Shawa et al. (2021)
Plasmid mediated	IncR plasmid With bla_NDM-1_	Azithromycin resistance	*E. hormaechei*	Doualla-Bell et al. (2021)
	*qnrA*, *qnrB*, *qnrS*	Quinolone resistance	Enterobacter spc.	Cano et al. (2009)
	*mcr*-9/10		Enterobacter spc.	Liao et al. (2022)
	*bla _NDM-1_* and *GES-5*		Enterobacteriaceae	Pedersen et al. (2018)

In this section, we have tabulated membrane proteins that play a role in the antimicrobial resistance mechanism of ESKAPEE strains. Preference was given to the membrane proteins and the plasmid encoded genes as they can be acquired through horizontal gene transfer. ESKAPEE pathogens account for majority of the nosocomial infections including catheter associated bloodstream and urinary tract infections, with *E. faecium* being one of them. *EfrAB* a heterodimeric transporter in *E. faecium*, confers resistance to antibiotics and biocides. Studies report that toxic substances like ethidium bromide, 4′, 6-diamidino-2-phenylindole (DAPI), doxycycline, novobiocin, doxorubicin amongst others were pumped out using the efflux pump activity of *EfrAB* (Davis et al., n.d.; Lubelski et al., 2007). Linezolid resistance arises as a result of inhibition of bacterial translation pathway (Diekema and Jones, 2000). A study suggests a plasmid encoding gene, *optrA* exhibiting resistance to phenicols and oxazolidinones (Wang et al., 2015).

Next, in the ESKAPEE list is *Staphylococcus aureus*, a superbug known to have developed resistance to nearly all the drugs of last resort. MRSA (Methicillin resistant *Staphylococcus aureus*) and VRSA (Vancomycin resistant *Staphylococcus aureus*), emerges as the most pathogenic strains due to the plasmid encoded *mecA* gene present on the mobile genetic element SCCmec (Jevons, 1961; Katayama et al., 2000; Olsen et al., 2006) and the presence of *vanA* operon (Arthur et al., 1993; Périchon and Courvalin, 2009) on the bacterial plasmid, respectively. Apart from these Gram-positive commensals, the Gram-negative bacteria have also turned out to be extremely resistant species responsible for high mortality rate worldwide. Through literature analysis, we enlist some membrane bound efflux pumps (Mehmood et al., 2020; Singh et al., 2022) and porin proteins (Chen et al., 2010; Singh et al., 2022) accountable for bacterial virulence and resistance to antibiotics belonging to carbapenem class of antibiotics against drug resistant *K. pneumoniae*. Further the problem is aggravated due to the biofilm forming capabilities of some of these pathogens. Studies report a few outer membrane proteins to be present at the helm of developing resistance and promoting biofilm formation in strains of Gram negative, *A. baumannii* (Labrador-Herrera et al., 2020; Nie et al., 2020).

Certain two-component regulatory systems also help in promoting virulence and outer membrane vesicles (Kim et al., 2019); which help the bacteria to adapt to the unfavorable environment conditions. Another harmful microbe, *P. aeruginosa* reportedly brings about resistance to macrolides and aminoglycosides via membrane proteins MphE, MsrE and a TCS *AmgS* (Ho-Fung Lau et al., 2013; Chen et al., 2020) respectively and via efflux pumps (*mexAB-oprM*, *mexAB-oprJ*; Azimi et al., 2015). Amongst the *Enterobacteriaceae* species they have conferred resistance through NDM beta-lactamases (Pedersen et al., 2018), *mcr* genes (Liao et al., 2022) and quinolone resistance genes (Cano et al., 2009). The last in the list is *E coli*, conferring kanamycin resistance through outer membrane mltA interacting proteinA (MipA) protein, i.e., responsible for interaction with MltA (membrane bound lytic transglycosylase) alongside TolC protein (Zhang et al., 2015). Also, AcrAB efflux pump with the New Delhi metallo beta-lactamase 1 (bla_NDM-1_) mediated resistance to carbapenems have been reported to be membrane proteins which can lead to potential drug candidates as specific targets (Chetri et al., 2019). Potentially both plasmid encoded and the membrane bound genes confers resistance to the bacterial species therefore, targeting these specific genes might serve to be an important drug candidate in overcoming the multi drug resistance problem.

## 5. Nanocarriers in overcoming AMR

Nanoparticles (NPs) represent alternatives in addressing AMR as not just drug delivery vehicles but also, they are capable of exerting inherent antimicrobial efficacy. Together they can generate an overall robust synergistic response (Vallet-Regí et al., 2019). Last few decades have seen significant applications in both disease diagnosis and therapeutics (Castillo et al., 2019). In terms of size, nanocarriers can range from 10 nm metal oxides of silver (silver NP or AgNP) and gold (gold NP or AuNP) to polymer-based nanoparticles 200–400 nm. Lipid NPs, nanoemulsions, polymer-lipid hybrid NPs or nanomicelles all represent myriad forms of nanocarriers which although differ in composition, can be utilized towards antimicrobial activity. Antibiotics can be conjugated to inorganic NPs or loaded (entrapped or adsorbed) onto organic NPs like liposomes or nanoemulsion. NPs can be tailor-made keeping in view the route of administration and the ensuing pathophysiological barrier- systemic through intravenous injection, aerosolized NPs for lung delivery, oral delivery for gastrointestinal infections (Kirtane et al., 2021). Nanocarriers can potentially improve the therapeutic index of encapsulated drugs or antibiotics by (1) Sustained or controlled release from nanocarriers and (2) minimizing the systemic drug concentration and consequently adverse effects. NP encapsulated drugs are protected from unwarranted enzymatic degradation or oxidation *in vivo* compared to their free drug counterparts (Moreno-Sastre et al., 2015).

NPs can exert their antimicrobial activity on the virtue of their surface charge, size and shape or a combination of all of these factors (Makabenta et al., 2020). Nanoparticles can potentially interact and interfere with proteins structure and integrity rupturing cellular membrane causing leakage and cell death. Antimicrobials-loaded nanoparticles, also referred to as “nanobiotics” can affect metabolic processes such as inhibition of transmembrane ATP formation, alter signal transduction, interfere with ribosomal subunits and their functioning, cause mitochondrial dysfunction and DNA damage in pathogens (Chakraborty et al., 2022a). In this review, however, as the cell wall/ cell membrane is the first line of bacterial defence system, the focus will be on the mechanisms of cell death based on interaction between nanoparticle and bacterial membranes.

### 5.1. Inorganic nanoparticles

Several inorganic nanoparticles are well established antimicrobial agents as well as drug delivery vehicles (Giner-Casares et al., 2016). Inorganic nanoparticles are used either in the form of metal like silver (Ag), gold (Au), zinc (Zn) or as metal oxide such as Zinc oxide (ZnO), Titanium oxide (TiO_2_) etc. Interestingly, akin to antibiotics, metallic nanoparticles can distinguish bacterial cellular system from eukaryotic cellular (mammalian cells) system through bacterial specific transport system and metalloproteins. But unlike antibiotics with specific mechanisms of action (against protein, ion channel, enzyme etc.), metal NPs are more efficient as they can mediate bactericidal activity through multiple mechanism such as membrane integration and damage, inhibition of drug efflux pumps, blocking electron transport, denaturing protein or mimicry and sequestering of endogenous ions (Lemire et al., 2013; Rumyantceva et al., 2019). This can have implications in breakdown of multiple pathways/ cellular components simultaneously leaving little or no time for bacteria to develop resistance mechanisms.

Therefore, metal-based nanoparticles present attractive antibiotic-alternatives against bacteria. Metal NPs such as Ag-NPs (Abbaszadegan et al., 2015), Au-NPs (Li et al., 2020), Cu NPs (Chatterjee et al., 2014; Sánchez-López et al., 2020), ZnO NPs (Kim et al., 2020; Vihodceva et al., 2021), αFe_2_O_3_ NPs (Vihodceva et al., 2021), TiO_2_ NPs (Siwińska-Stefańska et al., 2018) etc. have been reported extensively for their ability to either kill bacteria or to inhibit their proliferation.

Metal nanoparticles can be synthesized following sol–gel process, chemical vapor deposition (CVD) as bottom-up approach. Top-down approach include pyrolysis for synthesis of bimetallic NPs (Arora et al., 2020) or thermolysis for metal-polymer nanocomposites (Pal Singh Chauhan et al., 2019). Chemical reduction of metal salts is the most routinely employed technique for synthesis of metal NPs. Either strong reductants such as Sodium borohydride (NaBH_4_) and sodium hydrophosphite (NaH_2_PO_4_) or mild reductants such as plant extracts can be used as reducing agents for chemical or biological synthesis of NPs, respectively, (Kharissova et al., 2019). Furthermore, surfactants can be mixed to coat NP surface and to prevent aggregation. Examples of metal ions reducing to NPs include both noble and non-noble metals such as silver, gold, cobalt, nickel, copper and lead (Reverberi et al., 2016).

Inorganic NPs can be utilized either alone or with appropriate antibiotics for both additive or synergistic effect for pronounced bactericidal activity (Hari et al., 2014; Haji et al., 2022). Mahsa et al. conjugated antibiotic Vancomycin, which is a known inhibitor of cell wall synthesis to Ag NPs and evaluated against *E. coli* (MDR), *S. aureus* (Methicillin and vancomycin intermediate), *S. epidermidis* (MDR), *P. aeruginosa* (MDR) and *E. faecalis* (MDR, vancomycin resistant; Esmaeillou et al., 2017). Vancomycin-capped Ag NP treatment led to lower minimum inhibitory concentration (MIC) than free Vancomycin and exhibited overall stronger anti-bacterial activity against Gram positive than Gram negative bacteria. The authors suggested a synergistic effect of both Vancomycin and Ag NP in inhibiting cell wall synthesis and its structural disintegration.

Nanoparticles demonstrating robust properties in terms of small size (Suttiponparnit et al., 2011), high surface area to volume ratio and ease of preparation are suitable for theranostic purpose (Dadfar et al., 2019; Erkey and Türk, 2021). Due to having significant bactericidal activity, metal nanoparticle-based treatment of bacterial infections has attracted the interest of researchers to explore and understand it extensively (Yuan et al., 2017; Sánchez-López et al., 2020). While bactericidal activity is desirable, the widespread use of metal NPs is limited by its unwarranted accumulation and toxicity towards human tissues at high concentrations (Duval et al., 2019).

### 5.2. Organic nanoparticles

Organic nanoparticles referred to as polymeric or lipid-based nanoparticles are synthesized using natural or synthetic polymer, lipids, excipients as carrier constituents and acts as multifunctional delivery vehicles for antibiotics. Usage of organic NPs is growing exponentially due to their ability to deliver vast array of cargoes including- drugs, genes, peptides and other bioactive molecules. Additionally, organic nanoparticles are better than their inorganic counterparts owing to superior properties in terms of biocompatibility, biodegradability, enhanced cargo delivery with minimal systemic toxicity. Polymer-based nanocarrier can be further classified into polyester, polyamides etc. and have been known for effective delivery of antibacterial drug as well to targeted cells via pathway such as endocytosis, adsorption, ligand-receptor or contact-release etc. (Huh and Kwon, 2011; Ranjan et al., 2012). Methods of synthesis such as solvent diffusion, polymer precipitation, emulsion polymerization have been covered extensively for different class of organic nanomedicine such as lipid-based nanoparticles (Wang et al., 2020a), solid-lipid nanoparticles (Wang et al., 2020a), polymeric (Spirescu et al., 2021), lipid polymer hybrid nanocarriers (Shah et al., 2022).

There are various amphiphiles (Mehta et al., 2021), lipids (Fischer, 2020) and polymers (Qiu et al., 2020) which are known for exhibiting inherent antibacterial activity. This is especially important as the outer layer of nanocarrier is the first point-of-contact while encountering bacteria and can be tailor-made depending on target- in this case Gram-positive, Gram-negative or Mycobacterial cell wall. This can further enhance the functional activity of the nanoparticles. For example, Sarah et al., (Richards et al., 2018) recently showed that the polymer poly(dimethylaminoethyl methacrylate; PDMAEMA) having different degree of polymerization can act as antibacterial agent against *E. coli* and *M. tuberculosis* (model organism of TB). Mechanistic analysis further showed that in *E. coli* PDMAEMA can lead to cell membrane disruption followed by cell death whereas in *M. tuberculosis* it showed bacteriostatic effect.

In another recent work, Luo et al. prepared and characterized dialdehyde nanocrystalline cellulose (DNC) and evaluated in *S. aureus* (ATCC 6538P), *S. epidermidis* (ATCC 12228) and *S. pneumoniae* (ATCC 6305; Luo et al., 2021). Strong antibacterial activity against MRSA correlated with higher aldehyde content in DNC as observed *in vitro*. The bioactive nanomaterial significantly reduced MRSA infection on the skin of mice model, exhibited excellent skin compatibility, low cytotoxicity and no acute oral toxicity. The underlying mechanism was suggested to be disruption of membrane protein and leakage of cellular content. Harnessing such bioactive polymers (Wang et al., 2022b) or NP components can render nanocarrier additional bactericidal properties.

## 6. Mechanisms leading to bacterial death based on nanoparticle-membrane interaction

### 6.1. Interaction with cell wall

Cell wall is the outermost cellular component present in bacterial and archaeal species (Pasquina-Lemonche et al., 2020). The primary structural component of the cell wall comprises of peptidoglycan, which essentially provides structural integrity and acts as constraint to the integral turgor (Turner et al., 2014). Peptidoglycan is a macromolecule made of glycan units, crosslinked by peptide chain and therefore is a well-established crucial target of multiple antibiotics (Rojas et al., 2018). Several studies reported interaction of NPs with outer cell wall component led to breakdown of structural integrity of microbe followed by cell leakage and death.

In this context, shape of nanocarrier plays crucial role in its interaction with bacterial surface or biofilm. In a recent study (Acharya et al., 2018), shape dependent physical mutilation and lethal damage was observed in different bacterial species by sphere-shaped in contrast to rod-shaped silver NPs. AgNP-sp. showed enhanced antibacterial activity when exposed to bacterial strains- *E. coli* (MIC = 190 μg/ml), and *S. aureus* (MIC = 190 μg/ml), *B. subtilis* AST5-2 (MIC = 195 μg/ml), *P. aeruginosa* AL2-14B (MIC = 188 μg/ml) and *K. pneumoniae* AWD5 (MIC = 184 μg/ml). *K. pneumoniae* exhibiting the lowest MIC was further analysed using Field emission scanning electron microscopy (FESEM) and showed silver NP interaction with cell wall as the cause of bacterial death. The same group recently reported comparative evaluation of antibacterial activity of nanospheres with nanorods, nanotriangles and nanohexagons against Gram positive and Gram negative bacteria (Acharya et al., 2021). The authors attributed highest bactericidal effect in nanospheres owing to maximum release of silver ions over nanoparticles of other shapes.

Detailed analysis suggested that besides vancomycin, Ag NPs interacts with cell wall and rupture its architecture leading to cell death. In a similar work, instead of metal NP, chitosan-based polymeric NPs were formulated encapsulating ampicillin and tested against *S. aureus* strains (ATCC25923, ATCC29213, and ATCC43300; Ciro et al., 2019). It was observed that the positively charged chitosan in the outer NP layer interacted electrostatically with outer surface of bacteria specifically with lipoteichoic acid and ruptured its integrity followed by cell wall that led to loss of cellular integrity and cell death (Caudill et al., 2020).

### 6.2. Interaction with lipopolysaccharide

LPS is the outer most layer present in Gram negative bacteria. Functionally it imparts protection from foreign invaders and is involved in immune modulation. Cytoplasmic division requires the synthesis and transport of millions of new LPS molecules continuously (Clairfeuille et al., 2020). LPS comprises of lipid A membrane-anchor, core oligosaccharide and O-antigen (Shrivastava and Chng, 2019; Clairfeuille et al., 2020). Due to its immunostimulatory properties, LPS has been traditionally considered as potential target for antibiotics like polymyxin (colistin; Van Langevelde et al., 1998; Cano et al., 2009). Metals used in their nanoparticle form allow its absolute interaction with the LPS components and being smaller in size, are easy to bypass through channel proteins. Furthermore, the negative charge on bacterial LPS allows its electrostatic interactions with metal-based NPs and can disrupt membrane integrity (Beyth et al., 2015). Hence, these ionic interaction potentially generate oxidative stress via free radicals leading to cell membrane disruption followed by cell death (Lee et al., 2019; Mammari et al., 2022).

Khan et al. (2013) and Ansari et al. (2014a) studied the antibacterial activity of Ag NPs on 80 Gram negative clinical isolates of *E. coli* samples and investigated the mechanism of action behind therapeutic effect of NP. This investigation was confirmed by using Attenuated Total Reflectance-Fourier transform infrared (ATR-FTIR) spectroscopy. It was observed that IR spectra of membrane-based lipid polysaccharide (LPS) and Phosphoethanolamine (PE) changes significantly after exposure to Ag NPs. Moreover, it was also found that on one hand, the interaction of the O-antigen of LPS with Ag NPs leads to formation of hydrogen bond, on the other side, exposure of Ag NPs to membrane PE led to disruption of its phosphodiester bonds into phosphomonoesters and ultimately results in highly distorted alkyl chain.

**Figure 2:**
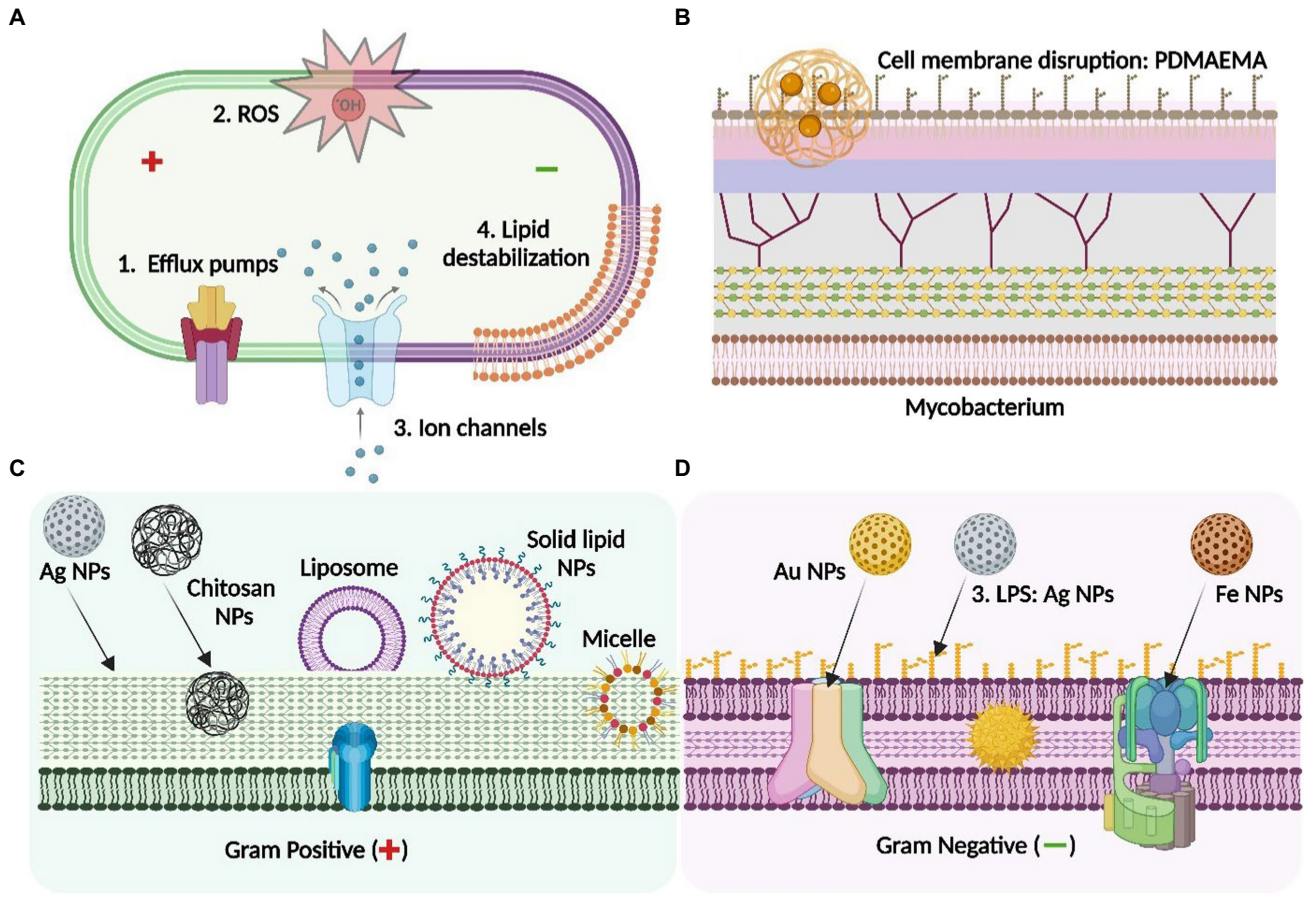
Antibacterial mechanisms of different class of nanoparticle: Mechanistic insight into the nanoparticle interaction with bacterial membrane to overcome AMR. **(A)** depicts the general membrane targets of the NPs through the activity of efflux pumps, reactive oxygen species (ROS), ion channels and membrane lipid destabilisation serving as antibacterial agents: **(B)** depicts the mycobacterial membrane and the interaction of PDMAEMA [poly(dimethylaminoethyl) methacrylate] polymeric NPs with the LPS layer of the mycobacterium, leading to cell membrane disruption; **(C)** represents the precise membrane interaction of the NPs with the Gram- negative bacteria- Ag NPs interact with LPS whereas AuNPs and Fe NPs binds with different targets through specific membrane transport proteins including the porin channels and the F0-F1 ATPase respectively; **(D)** shows the specific targets of different types of NPs on the Gram-positive bacterial membrane-Ag NPs, chitosan (polymeric) NPs, solid-lipid NPs: These interact with peptidoglycan layer of the cell envelop to exert antimicrobial activity. Others, including micelle or liposome-based NPs serves as a carrier to enhance the penetration of the antibiotic by interacting with the thick peptidoglycan of the bacterial membrane. Figure created in Biorender.

Another similar study has shown that exposure of Al_2_O_3_ NPs to clinical isolates of *E. coli* significantly inhibited the proliferation of bacterial population. Investigation of antibacterial mechanism as observed by ATR-FTIR, suggested that interaction of membrane phospholipid especially L-α-Phosphoethanolamine (PE) and O-antigen of LPS with Al_2_O_3_ NPs led to the disruption of cellular integrity. Additionally, formation of H-bond, interfering with membrane ligands led to change in the amphiphilicity of membrane and hence caused cell leaking and cell death (Ansari et al., 2014b; Figure 2).

### 6.3. Interaction with membrane proteins of bacteria

Bacterial membrane is considered as one of the most prominent targets for any anti-bacterial drug or antibiotic as the outer structure acts as a physical barrier and mediator between outer environment and inner cellular components. The membrane proteins and their components including transport protein (Li et al., 2016), anchor proteins (Sun et al., 2021), structural proteins (Lyu et al., 2022), efflux proteins (Soto, 2013; Sun et al., 2014; Du et al., 2018) and ion channels (Prindle et al., 2015) play essential role towards cell physiology and metabolic regulation. Outer leaflet of plasma membrane has architecture of LPS while inner leaflets incorporate mixture of approximately 25 phospholipid type and various protein complexes too. Many metals in nanoparticulate form can exert their antimicrobial activity by destabilizing these membrane proteins (Rudramurthy et al., 2016).

Hamida et al. (2020) showed that biogenic Ag NPs cause the bacterial cell death via the generation of ROS and experimental result concluded that exposure of Ag NPs show biofilm inhibition and virulence activities in MRSA. Mechanical analysis suggested that disruption of membrane proteins mediated via several mechanisms including generation of free radicals leading to oxidative stress, lipid peroxidation interferes with fluidity and stability of membrane leading to cell death. In another recent study, Jiang et al. (2022) exhibited that when nanoparticle-pinched polymer brushes (NPPBs) consisting chemically inert silica nanospheres (covalently grafted with hydrophilic polymer brushes) were exposed to Gram negative strains including *E. coli*, tobramycin and gentamycin-resistant *P. aeruginosa* PA14, and Gram Positive *S. aureus* and *S. aureus* MU50 (methicillin, oxacillin, and vancomycin-resistant *S. aureus*), it induced pore formation in the membrane followed by cell death (Nikaido, 2009). Although it was suggested that smaller sized NPPBPs had more intensified antibacterial potential against both Gram positive and Gram negative bacteria compared to larger NPPBs (dsilica > 50 nm).

Gabrielyan et al. (2019) noted antibacterial effect of Fe_3_O_4_ NPs on two resistant strains of Gram negative bacteria- ampicillin resistant *E. coli* DH5α-pUC18 and kanamycin resistant *E. coli* pARG-25 strains, respectively, and further evaluated the mechanism of action. It was observed that Fe_3_O_4_ NPs, although in a concentration dependent manner, significantly started reducing the bacterial growth and increased the latent lag phase at the concentration of 50 μg/ml and exhibited maximum inhibition at concentration of 250 μg/ml. Although kanamycin resistant *E. coli* pARG-25 was found to be more susceptible than ampicillin resistant *E. coli* DH5α-pUC18 but exposing 100 μg/ml of Fe_3_O_4_NPs to *E. coli* DH5α-pUC18 either in the absence or presence of antibiotic ampicillin or Kanamycin decreased the cell viability by 5- to 7-fold, respectively. They further determined H^+^ flux and H^+^ membrane conductance as an indicator of bacterial membrane function. The production of H_2_, interestingly suggested that Fe_3_O_4_ decreased energy dependent H^+^ efflux by *E. coli* DH5α-pUC18 by ~1.2 and ~1.5-fold, respectively, when grown in the absence and presence of ampicillin. Interestingly, exposure of Fe_3_O_4_ to *E. coli* grown in the absence of antibiotics led to ~1.7-fold decrease in H^+^ flux in the presence of N,N-Dicyclohexylcarbodiimide (DCCD), inhibitor of F0F1 ATPase (Vardanyan et al., 2015). Besides this, approximately ~1.2-fold H^+^ conductance of *E. coli* was enhanced, in the absence of ampicillin while in the presence of ampicillin ~1.9 fold increased when compared to control. Lastly, they estimated the H_2_ production, and observed that besides reducing the redox potential (Eh) to negative value, H_2_ production was also reduced (Poladyan et al., 2012; Sargsyan et al., 2016).

Trchounian et al. (2017) has reported that H_2_ production is directly related to the membrane associated formate hydrogen lyase (FHL) complexes that functions to split formate into H_2_ and CO_2_. Post exposure to Fe_3_O_4_ (in absence of antibiotic), it was observed that H_2_ production was decreased ~1.2-fold as compared to control. Through this work, including decrease in H^+^ fluxes in the absence of FoF1 ATPase author concluded that Fe_3_O_4_ NPs potentially interact with membrane associated proteins including FHL, membrane transport channel protein etc. which ultimately distort the membrane integrity by enhancing the membrane permeability and leads to cell death.

### 6.4. Efflux proteins

Beside targeting global membrane proteins, NPs can also exert bactericidal effect upon interaction with particular protein present in the membrane. We found very few studies to have evaluated specific NP-membrane protein interaction, and these have been included in this review. Efflux pumps are transport proteins which essentially play role in extrusion of substances from inner to exterior environment. In bacterial system, at least 6 different drug efflux pumps have been identified that contribute towards the efflux pathways (Du et al., 2018). Among them, there is one ATP-binding cassette (ABC) which directly utilises ATP as an energy source to drive transport, while rest type of efflux pump known as secondary active transporters including major facilitator superfamily (MFS), the multidrug and toxin extrusion (MATE) family, the small multidrug resistance (SMR) family, the resistance-nodulation-cell division (RND) superfamily and the proteobacterial antimicrobial compound efflux (PACE) etc. empowered by electrochemical energy generated in membrane ion transport gradient (Hassan et al., 2013, 2015).

Interestingly, NPs can easily bypass efflux pumps by two mechanisms – (1) act as Trojan horse and deliver antibiotics intracellularly and (2) interact with efflux pump causing irreversible blockage. Several studies have reported effective antibacterial activity when bioactive molecule(s) delivered by encapsulating into nanoparticles. For example, Padwal et al. (2014) and Padwal et al. (2015) studied the antibacterial effect of iron oxide functionalized by polyacrylic acid in the presence of rifampicin antibiotic (PLA-MNP). Exposing *Mycobacterium smegmatis* to the combination of rifampicin and PLA-MNP led to 4-fold reduction in bacterial proliferation as compared to either of the treatments. Further mechanistic analysis suggested that beside enhanced cell permeability, efflux pump disruption was responsible.

In another study, it was observed that Copper Nanoparticles (Cu NPs) exhibited bactericidal activity by inhibiting *norA* pump through generation of Cu which directly blocked the efflux pump (Ashajyothi et al., 2016). Similarly, Au NPs functionalized with pyrimidine showed antibacterial activity by sequestering ions such as magnesium and calcium present in the cell. Further analysis suggested that use of vancomycin and Au NPs together led the inhibition of *E. coli* and *P. aeruginosa* proliferation by directly targeting efflux pump (Zhao et al., 2010).

In another study, Christena et al. (2015) showed that Cu NPs have potential to act as antibiofilm inhibitor as well as the efflux pump inhibitor. Study revealed that exposure to Cu NPs (0.065 mM) exhibited remarkable inhibition in wild type strains of both *S. aureus* and *P. aeruginosa* and less but significant efflux inhibitory effect against MRSA and drug resistant mutant strains of *S. aureus*. Exposure to and increased concentration of 0.13 mM Cu NPs led to significant inhibition of biofilm formed by *S. aureus* and *P. aeruginosa*. Efflux pump inhibition and antibacterial effect of Cu NPs is suggested partly by particles’ effects and ionic effects, respectively. In support of their hypothesis on efflux pump inhibition, the authors performed cartwheel assay, real time efflux and membrane permeable related studies.

In another recent study, it was shown that the magnetite nanoparticles (MNP) coated with polyacrylic acid (PAA) treated together with rifampicin (a first line anti-TB drug) exhibited synergistic effect including 4-fold growth inhibition of *M. smegmatis* mc2 155 and 3-fold enhanced intracellular aggregation of rifampicin. Through mechanistic study, it was concluded that MNP-PAA formulation blocked the efflux system of mycobacterium (Padwal et al., 2014). These studies suggest that combination of inorganic NPs together with antibiotics can be a promising strategy in blocking efflux pump inhibitors and deliver antibiotic dose within pathogens at therapeutic levels.

### 6.5. Ion channels

Ion channels are the membrane component that play vital role in maintaining physiological balance and homeostatic condition. Different types of ion-specific channels have been identified based on voltage gated and non-voltage gated. Membrane ion channels like Na^+^, K^+^ and Cl^−^ are well characterized at structural and functional level (Booth et al., 2003). Altogether, ion channel functions include uptake of particular ions and small molecules etc. (Booth et al., 2003). Upregulation of membrane ion channels have been reported in MDR bacteria (Nikaido, 2009). Interestingly, several studies have suggested good affinity of NPs towards ion channels either through ionic or electrostatic interaction (Yin et al., 2019).

Au NPs functionalized with pyrimidine (Au-DAPT) 4,6-Diamino-2-pyrimidinethiol experimented clinical isolates, MDR *E. coli* and MDR *P. aeruginosa* hospitals in China showed effective bacterial cell death. Further, mechanistic analysis suggested that Au-DAPT interact with outer membrane vesicles (OMV) especially sequester and chelate the ions Mg^2+^ and Ca^2+^ which caused disturbance in membrane potential, enhancement in cell membrane permeability and thereby cell death (Zhao et al., 2010). Similarly, Song et al., found that exposing Chlorohexidine nanoemulsion against MRSA caused K^+^, Mg^2+^ ions leakage which increased the membrane electrical conductivity and thereby exhibited strong ability to damage membrane proteins and increased overall cellular permeability (Song et al., 2016).

### 6.6. ROS-mediated bacterial killing

ROS is a stress response wherein reactive oxygen species generated using photosensitizers (photosensitive compounds/ metals) which interacts with bacterial biomolecules such as lipids, fatty acids, proteins, DNA, ribosomes etc. eventually leading to cell death (Hong et al., 2019; Li et al., 2021). Although microbes possess antioxidant system to maintain or neutralize the cellular ROS response but an excessive ROS generation can potentially disrupt cellular homeostasis. Nanomaterials having potential to interact with biomolecules, proteins or other macromolecules generate ROS through lipid peroxidation, generation of free radical (^1^O_2_), superoxide radicals (O^2−^), and hydroxyl radicals (^.^OH). Nanomaterials especially certain metal-based NPs (such as Copper, Tellurium, Titanium) are known to cause ROS development owing to pro-oxidant functional group which allow more ultra-reactive surface, secondly, the involvement of transition metal ion nanoparticles providing multivalent sites for interaction and after that, post-internalisation interaction of NPs with cellular components (Chakraborty et al., 2022b).

Several studies have shown promising antibacterial activity due to ROS generation. Alhadrami and Shoudri (2020) studied the effect of TiO_2_ NPs on MRSA, *E. coli, and P. aeruginosa*. The authors observed effective ROS generation with TiO_2_ due to interaction with membrane lipid and proteins leading to cell death. Similarly, Pramanik et al. (2012) showed that exposure of Copper Iodide (CuI) to *Bacillus subtilis* (ATCC 6633)*, Shigella dysenteriae* (ATCC 12039) and *E. coli DHF 5α (*ATCC 10536) cause ROS generation. Further, mechanistic analysis concluded that interaction of Copper Iodide with various amine groups present in the membrane biomolecules and others led to generation of ROS causing cell death. In another study, Sarwar et al. (2016) evaluated the activity of ~10 nm ZnO NPs on two biotypes of O-serotype of *V. cholera* -Classical and El Tor, with the latter being more susceptible in biofilm and planktonic forms. Further analysis showed enhanced membrane fluidity and polarization along with protein leakage. This distortion of membrane integrity was caused by ROS-mediated free radical generation and its interaction with membrane lipids, proteins, fatty acids followed by cell death (Sarwar et al., 2016). In another recent study, Morena et al. (2021) showed the generation of ROS due to exposure of Hybrid Tellurium Lignin Nanoparticles (TeLigNPs) in *S. aureus* (ATCC 25923), *E. coli* (ATCC 25922), and *P. aeruginosa* (ATCC 10145). Further mechanistic analysis suggested interaction of active surface of TeLigNPs with hydrophilic surface of bacterial membrane. The NPs further integrated with the outer membrane causing lipid peroxidation, generation of highly reactive short aldehyde chain which diffused intracellularly to oxidize amine and thiol groups of proteins thereby affecting the cellular homeostasis and causing cell death (Vermaas et al., 2019).

ROS generation using photosensitizer and light under particular wavelength has successfully translated as research field referred to as antibacterial photodynamic therapy (aPDT). Recently, Yan et al. (2021) designed ultra-thin hollow silica-chitosan NPs and loaded with photosensitizer Ce6. In comparison to free Ce6, its nanoparticle formulation led to stronger antibacterial activity against *S. aureus*, enhanced adherence and obliteration of mature *S. aureus* biofilms and 81% decline in biomass. In another work, non-toxic plant pigment chlorophyll was demonstrated to exhibit excellent photodynamic bactericidal properties (Liu et al., 2022). Additionally, if combined with antibiotics, aPDT can show synergistic effects as demonstrated by Liu et al. (Wang et al., 2022a) with gentamycin (antibiotic) and Toluidine (photosensitizer) in terms of growth inhibition in MDR *S. aureus*. The authors also reported the treatment to be effective in burn-infected mice by reducing number of bacteria colonizing the wound, decline in inflammatory markers and promoting wound healing process. Co-encapsulating photosensitizers with antibiotics with NPs for synergistic effect should be explored for nanomedicine-based aPDT (Table 2).

**Table 2:** Nanoparticles and their mechanism of action at the NP-bacterial membrane interface.

S. No.	Nanoparticle	Investigated bacteria	Interaction with/membrane target	Mechanism of nanoparticle action	Reference
1.	Ag NPs	*E. coli*	LPS and L- α -phosphatidyl-ethanolamine (PE)	NP interacted with O-antigen part of LPS via hydrogen bonding Ag NP broke phosphodiester bond of PE into phosphate monoesters to form highly disordered alkyl chain	Ansari et al. (2014a)
2.	Al_2_O_3_ NPs	*E. coli*	LPS and L- α -phosphatidyl-ethanolamine (PE)	LPS binding to Al_2_O_3_ NPs through hydrogen bonding and ligand exchange structural changes in phospholipids led to loss of amphiphilic properties	Ansari et al. (2014b)
3.	Au NPs functionalized with branched polyethylenimine	*B. subtilis*	Teichoic acid	Electrostatic interaction between NPs and teichoic acid No interaction with mutant having teichoic acid but lacking alanine	Caudill et al. (2020)
4.	Ampicillin- chitosan–polyanion nanoparticles	*S. aureus* strains (ATCC25923, ATCC29213 and ATCC43300)	Lipoteichoic acid (LTA)	Electrostatic interaction between chitosan and LTA leads to disturbance in membrane homeostasis MIC for free and NP-encapsulated Ampicillin was 0.26 μg/ml and 0.13 μg/ml respectively	Ciro et al. (2019)
5.	Curcumin-functionalized poly(lactic-co-glycolic acid)-dextran micelles	*P. putida* (PCL 1482) and *P. fuorescens* (PCL 1701) biofilms	Exopolysaccharide (EPS)	Micelle possibly altered surface hydrophobicity in bacteria Disruption of established biofilms induced electrostatic interaction between micelles and EPS to weaken overall architecture	Barros et al. (2021)
6.	CTAB-coated gold nanoshell	*S. aureus, E. coli, S. enterica* and *P. aeruginosa*	Cell wall	The aim was to use gold nanoshells as sensors for bacterial detection Both enzyme β-galactosidase and bacteria competed to interact with gold nanoshells Electrostatic interaction with LPS led to formation of gold nanoshell aggregates in cell wall causing cell death	Tanvir et al. (2017)
7.	Spherical and Rod-shaped Ag NPs	*E. coli* (ATCC25922) and *S. aureus* (ATCC25923). *B. subtilis* (AST5-2), *P. aeruginosa* (AL2-14B32) and *K. pneumoniae* (AWD5)	Cell wall	FESEM analysis suggested rupture of cell wall Rod-shaped Ag NPs showed enhanced antibacterial activity	Acharya et al. (2018)
8.	Curcumin loaded Solid Lipid Nanoparticles	*E. coli* (ATCC25922) *S. aureus* (ATCC25923)	Cell permeability	Combination of cholesterol-curcumin exhibited stronger antibacterial activity and led to enhanced cell membrane penetration and leakage	Jourghanian et al. (2016)
9.	Triclosan-loaded micellar nanocarriers	*S. aureus* (ATCC12600GFP) and bioluminescent *S. aureus* Xen36	Cell permeability	Enhanced biofilm penetration of micelle and accumulation due to electrostatic interaction with bacterial cell surface at acidic pH Triclosan release due to micelle degradation by bacterial lipase	Liu et al. (2016)
10.	Graphene oxide Ag Nanocomposite	*Enterobacter cloacae* *Staphylococcus mutans*	Cell leakage	Protein leakage was assessed and found to be significant in Gram – than in Gram + Gram + has thicker cell wall and posed barrier to nanocomposite penetration	Kulshrestha et al. (2017)
11.	Au NP capped with pyrimidine (Au-DAPT) 4,6-Diamino-2-pyrimidinethiol	MDR clinical isolates- *E. coli* and *P. aeruginosa*	Membrane Ions: Mg^2+^ and Ca^2+^ ion of outer membrane vesicle (OMV)	Sequestration or chelation of Mg^2+^ and Ca^2+^caused by Au-DAPT lead to the disruption of membrane integrity which lead to leakage of cellular components	Zhao et al. (2010)
12.	Poly(acrylic acid; PAA)-coated iron oxide (magnetite) nanoparticles (PAA-MNPs) and Rifampicin (TB drug)	*Mycobacterium smegmatis*	Efflux pump	Iron oxide NPs acted as efflux pump inhibitor which resulted in up to a 3-fold-increased accumulation of rifampicin inside Mycobacterium	Padwal et al. (2014)
13.	Cu NPs	Wild type- *S. aureus* and *P. aeruginosa*. MRSA and drug resistant mutant- *S. aureus*	Efflux pump	Exhibited remarkable efflux inhibition activity Reverse the MIC of the mutant *S. aureus* strain for ciprofloxacin by 4-fold	Christena et al. (2015)
14.	CuI NPs	*B. subtilis* (ATCC6633) and *E. coli DHF 5α* (ATCC10536)*, Shigella dysenteriae* (ATCC12039)	ROS generation	Bactericidal activity due to ROS formation of the surface of CuI NP due to interaction with amine functional group of various biomolecules on cell membrane	Pramanik et al. (2012)
15.	TiO_2_ NPs	*MRSA, E. coli* and *P. aeruginosa*	ROS generation	NP interaction with membrane protein and lipid leads to generation of ROS followed by cell death	Alhadrami and Shoudri (2020)
16.	ZnO NPs	*Vibrio cholera*	ROS generation and membrane disruption	NPs increased fluidity and depolarization of membrane, protein leakage leading to bacterial death	Sarwar et al. (2016)
17.	Chlorohexidine acetate nanoemulsion (CNE)	Skin burn wound MRSA infection	Ion leakage and membrane disruption	CNE treatment led to leakage of K^+^, Mg^2+^ Ions, DNA and protein Increase electrical conductivity and disruption of cell wall and cell membrane	Song et al. (2016)
18.	Hybrid Tellurium−Lignin Nanoparticles (TeLigNPs)	*S. aureus* (ATCC25923), *E. coli* (ATCC25922), and *P. aeruginosa* (ATCC10145),	ROS generation and membrane disruption	Strong antibacterial activity due to interaction of lignin with hydrophilic surface of Gram – bacteria as compared to Gram + Insertion of TeLig NPs into the outer membrane caused lipid peroxidation decomposing it into highly reactive short-chain aldehydes which further diffused into cytoplasm and oxidize thiol and amino groups of proteins leading to death	Morena et al. (2021)

## 7. Antimicrobial nanomedicine: pre-clinical results

Overall, nanomedicine has shown promising results in terms of bench-to-bedside research for cancer therapy and these innovations were responsible for early FDA approvals for Doxorubicin-loaded liposome (Doxil®) and Paclitaxel-loaded albumin nanoparticles (Abraxane®) in early 1990s. However, approvals for antimicrobial nanomedicine are few and far between till date. While silver nanoparticles have made it to marketed products from antibacterial creams to face masks, only a handful other NPs are approved as antimicrobial agents, most of which belong to liposomes. Liposomes represent small- (SUVs) or large-unilamellar vesicles (LUVs) composed of phospholipid bilayers with the ability to entrap both hydrophilic and hydrophobic compounds and ease of surface functionalization (Ferreira et al., 2021). They present the most widely researched and FDA approved nanomedicine (Table 3) due to their stability, fusablity with bacterial membrane (fusogenic liposomes) and enhanced biological performance in terms of controlled antibiotic delivery due to prolonged plasma circulation. Besides systemic administration, in one of the recent clinical trials, liposomal amikacin for inhalation (LAI) for nebulizer-based inhalation in *Mycobacterium abcessus* infected Cystic Fibrosis patients showed promising results (Caimmi et al., 2018).

**Table 3:** FDA Approved antimicrobial nanomedicine.

Name	Active Ingredient	Bacteria/ Infection	Company
Arikayce®	Amikacin	Mycobacterium avium complex (MAC) lung infection	Insmed Inc.
ArikaceTM	Amikacin	*P. aeruginosa*, Cystic Fibrosis	Transave Inc.
LipoquinTM (fast release) and PulmaquinTM (slow release)	Ciprofloxacin	Non-cystic fibrosis bronchiectasis (*P. aeruginosa*)	Aradigm
CAF01	Subunit protein antigen Ag85B-ESAT, DDA, TDB	Tuberculosis	Statens Serum Institute
ALIS	Amikacin	Nontuberculous Mycobacterial Lung infection	Insmed Inc.

Other ‘nanobiotics’ in clinical use include formulations of mupirocin, gentamicin and polymyxin B. Recently, during the second wave of Covid, patients recovered from long Covid developed secondary infection due to the fungus Mucormycosis (Black fungus). Amphotericin is an anti-fungal agent against mucormycosis but is highly toxic to nervous system and is administered as Ambisome®- liposomes encapsulating Amphotericin. There was a global shortage of the liposome due to increase in number of patients with Mucormycosis. Hence, it is imperative to develop antibacterial nanomedicine as humanity wades through the surge of AMR.

Mechanistic insights into membrane interaction with NP can yield potential targets for specific action only to bacteria without affecting human cells. Development of such nanoparticles for active delivery can further improve antibiotic pharmacokinetics. There is need to develop effective strategy to counter life threatening situation such as non-response to antibiotics or sepsis. Administering nanoparticles which can block efflux pump inhibitors in such critical situations can be specifically helpful as they can further facilitate antibiotic retention and bacterial killing of resistant strains. Antimicrobial nanomedicine, especially metal-based NPs have shown quick response in killing bacteria upon interaction with bacterial membrane. In general, translational research on nanocarrier-based antibiotic delivery should be promoted as it can yield faster antibacterial response at lower antibiotic doses. Additionally, it prevents undesirable systemic exposure of antibiotics to non-target human tissues thereby reducing the development of microbial resistance.

Generation of toxic by-products following treatment with nanoparticles is a major roadblock in advancement of nanomedicine. Toxicitiy of Ag, ZnO, and CuO NPs and the ensuing toxicity in organs such as spleen, liver, lungs, bone marrow and colon owing to tissue accumulation have been reviewed previously (Liu et al., 2020). Others have reported neuro- and nephrotoxicity with Al_2_O_3_ and CuO NPs, respectively, due to DNA damage and oxidative stress (Ivask et al., 2014; Liu et al., 2020). Metal-NPs toxicity can be mitigated by optimizing and administering minimum dose or combination with antibiotics to obtain synergistic effects. Additionally, coating with aptamers, antibody can improve therapeutic effect by targeting specifically to bacteria. Alternatively, organic NPs formulated with biodegradable carrier moieties such as polymers, lipids or amphiphiles can alleviate toxicity issues (Fischer, 2020; Qiu et al., 2020; Mehta et al., 2021).

Besides addressing toxicity and improving pharmacokinetics *in vivo*, the clinical translatability of nanomedicine in terms of batch-to-batch reproducibility, contaminant-less synthesis and storage stability is pertinent. Improved quality control guidelines for industry-grade nanomaterials and intellectual property regulations can further bring nano-based products through clinical trials to consumers.

## 8. Conclusion and future perspectives

As newer pathogenic strains emerge, development of alternative antimicrobial therapy is imminent. Using nanoparticles as a vehicle for delivering therapeutic agents such as antibiotics or antimicrobial peptides not only enhances its delivery efficiency but also reduces drug toxicity, improves bioavailability and specificity. Nanoparticles have shown ability to penetrate biofilms and can also act as slow-release reservoirs of antibiotics. However, most studies are restricted to *in vitro* bactericidal activity without evaluation of mechanism of action. Comprehensive studies are required to understand the mechanism of interaction of different classes of nanoparticles with bacterial membrane. Research should focus on narrowing down the generic NP-membrane interaction mechanisms, as the nanoparticle interaction can differ depending on the structural differences of Gram negative, Gram positive or Mycobacterium membranes.

Furthermore, optimizing nanoparticle formulations for physical and chemical stability, batch-to-batch reproducibility, quality control and scale up capabilities are paramount to move towards *in vivo* testing. This gap needs to be further abridged by additional studies of ‘antibacterial nanomedicine’ on mice model of bacterial infection and biodistribution profile to aid its clinical translatability. Various robust animal models of skin, lung, gut and systemic infections exist and must be utilized to further characterize and test antimicrobial nanomedicine.

In this review, we highlighted on the bacterial membrane where most of the AMR-related alterations occur at genomic or functional level. This can have direct implications in the interaction of nanoparticles at the bacterial membrane interface. Although few, we also reviewed research articles that elucidate mechanism of NP-membrane interactions. Identification and characterization of potential bacterial membrane targets specifically in the drug resistant strains can help design NPs towards targeted therapy. Surface conjugation with aptamers or antibodies can provide specificity and further reduce the dose of antibiotic required for antibacterial action. With few clinically approved nanomedicine, there is scope for major research in utilizing the potential of nanotechnology as an alternative to antibiotics.

## Author contributions

MSe and MSi: conceptualization, writing—review and editing, and supervision. SM and SG: writing—original draft preparation. All authors contributed to the article and approved the submitted version.

## Conflict of interest

The authors declare that the research was conducted in the absence of any commercial or financial relationships that could be construed as a potential conflict of interest.

## Publisher’s note

All claims expressed in this article are solely those of the authors and do not necessarily represent those of their affiliated organizations, or those of the publisher, the editors and the reviewers. Any product that may be evaluated in this article, or claim that may be made by its manufacturer, is not guaranteed or endorsed by the publisher.
